# Estimation and Correction of the Partial Volume Effect Using Personalized Phantoms of Lymph Node Metastases in Lutetium 177 SPECT/CT Dosimetry

**DOI:** 10.1007/s11307-026-02103-x

**Published:** 2026-04-29

**Authors:** J. R. Hinz, T. Kuwert, M. Beck, P. Ritt, A. Grings

**Affiliations:** 1 Department of Nuclear Medicine, Uniklinikum Erlangen, Ulmenweg 18, 91054 Erlangen, Germany; 2 Chair of Nuclear Medicine, Friedrich-Alexander-Universität Erlangen-Nürnberg (FAU), Erlangen, Germany

**Keywords:** Dosimetry, Lu-177, Lymph node, Tumor dosimetry, SPECT, PVE, Phantom, 3D-printing

## Abstract

**Purpose:**

This study aims to quantify and to correct for partial volume effect (PVE) in lymph node metastases of prostate cancer (PC) on Lu-177 SPECT images using 3D-printed phantoms of these structures to establish a ground truth.

**Procedures:**

Ten individuals with clearly delineated, SPECT-positive lymph node metastases were retrospectively selected from a cohort of PC patients undergoing radioligand therapy (RLT). Manual segmentation of the metastases was performed on the CT component. The segmented lymph nodes were 3D-printed as patient-specific lymph node phantoms with volumes ranging from 0.18 to 23.7 ml using a high-resolution 3D printer. These phantoms were filled with Lu-177 and analyzed in a SPECT/CT system to quantify PVE. An exponential curve fit of the recovery coefficient (RC) versus the surface-area to volume ratio (SA:V) was derived from the spheres of a NEMA ICE body phantom and used to correct for PVE in the lymph node phantoms. This method was compared with other post-reconstruction partial volume corrections (PVC).

**Results:**

For a tumor to background ratio (TBR) of 10:1 the RCs varied widely, from 82% to 10.4% depending on phantom size. Using the NEMA IEC body phantom, an exponential correlation was established between SA:V and RC, with R^2^ values exceeding 0.97 across measurements at four different TBRs. Using this curve for PVE correction, the RCs of the lymph node phantoms had an average deviation from the ground truth of 1.15 ± 10.15% (average ± standard error of the mean). This method had a higher accuracy than the other PVCs studied.

**Conclusions:**

The low RCs obtained for lymph node metastases suggest that Lu-177-dosimetry is in these structures grossly inaccurate without PVE correction. Applying the SA:V/RC curve established using the NEMA IEC body phantom for this purpose reduces PVE-related errors considerably.

**Supplementary Information:**

The online version contains supplementary material available at 10.1007/s11307-026-02103-x.

## Introduction

Prostate cancer is the second most common malignancy among men worldwide [1]. Since most bone, visceral, and lymph node metastases overexpress the prostate-specific membrane antigen (PSMA) [2, 3], targeted radionuclide therapy (RLT) has become a standard therapeutic approach for metastatic castration-resistant prostate cancer (mCRPC) [4–6]. Currently, the most widely used radionuclide is the β-emitting lutetium (^177^Lu), administered in the form of [^177^Lu]Lu-PSMA-617 or [^177^Lu]Lu-PSMA-I&T [7, 8]. According to the results of the VISION trials, standardized activity levels are used in clinical practice [9, 10]. From a patient-centered perspective, this approach can lead to overtreatment, resulting in unnecessary hematotoxicity. Conversely, in cases of high tumor burden or low PSMA expression, undertreatment may occur, leading to insufficient antitumor effects [11]. Adjusting administered activity doses based on patient-specific tumor dosimetry can potentially optimize therapeutic outcomes [12]. Tumor dosimetry is performed based on post-therapeutic Lu-177 imaging using SPECT/CT [13]. Patients eligible for RLT predominantly present with lymphogenic or osseous metastases, with visceral metastases being less common but clinically significant when present [10]. Bone metastases present disadvantages regarding tumor dosimetry. Clear visual delineation and accurate volumetry of bone metastasis can be challenging due to limited spatial resolution and tissue-/density- related effects in quantitative SPECT/CT [14, 15]. In contrast, lymph node metastases (LNM) are more suitable for tumor dosimetry, as their relatively homogeneous soft-tissue density allows for more reliable activity quantification and volumetric assessment [16–18]. However, LNM are usually relatively small in size. A significant challenge for accurate dosimetry in small structures such as lymph nodes is the partial volume effect (PVE). This effect arises from the limited spatial resolution of SPECT systems, particularly for structures smaller than approximately 2–3 times the full width at half maximum (FWHM) and can cause a significant underestimation of radionuclide concentration [19, 20]. The extent of the partial volume effect is influenced by numerous factors [20]. A key determinant is the spatial resolution of SPECT devices, which is primarily limited by the performance of the collimator [19]. In addition to technical specifications, the characteristics of the structure under examination also affect the PVE. These include size and shape [21], the surface-to-volume ratio (SA:V) [22], and the activity in the surrounding tissue [20]. The spatial resolution of SPECT systems used for ^1^⁷⁷Lu imaging typically ranges from 10 to 20 mm FWHM, depending on the collimator design, acquisition geometry, and reconstruction protocol [23, 24]. Inadequate correction for the PVE leads to a significant underestimation of the radiopharmaceutical concentration, which skews dose estimation substantially [23, 25]. Thus, partial volume correction (PVC) is critical for accurate dose estimation in targeted radionuclide therapy, particularly for smaller metastases such as LNM. Several methods and approaches for PVC have been explored in research, which can be categorized into reconstruction-based and post-reconstruction-based techniques. Despite the advantages of reconstruction-based approaches, their adoption in clinical practice remains limited due to the requirement for customized reconstruction algorithms. Therefore, our study focuses on post-reconstruction-based methods, which are more commonly employed in clinical settings due to their simpler implementation and quantification [26]. This study aims to quantify the extent of PVE in lymph node metastases through the analysis of patient-specific LNM phantoms and to evaluate the potential of various PVC methods to optimize dosimetry for small structures. This approach could enable the generation of more reliable dosimetry data for small metastases, such as LNM, in the future, further individualizing therapies for patients with metastatic castration-resistant prostate cancer.

## Materials and Methods

### Patient Selection

We retrospectively reviewed a cohort of 63 patients with metastatic castration-resistant prostate cancer who underwent radioligand therapy with [^177^Lu]Lu-PSMA-617, including routine multi-time-point (MTP) dosimetry imaging at 0 h, 4 h, 24 h, 48 h, and 72 h post injection between February 2021 and March 2023. Patients were included if they presented with clearly delineated, SPECT-positive, and segmentable lymph node metastases. Based on these inclusion criteria, a total of ten patients were selected for personalized phantom fabrication.

### CT in Lu-177 SPECT/CT Dosimetry

**Fig. 1:**
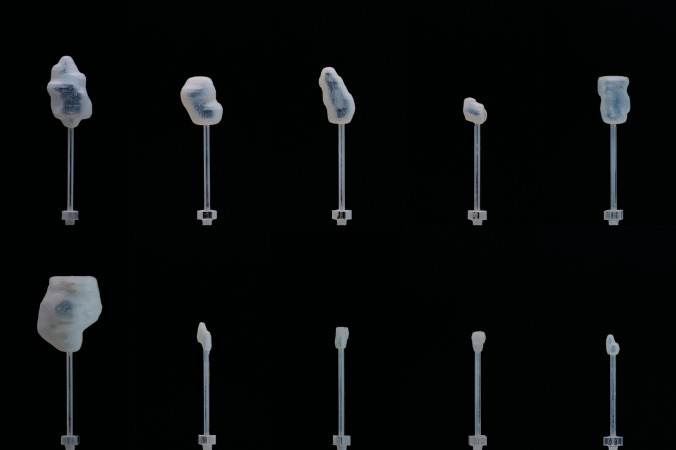
The 10 3D-printed lymph node phantoms used for validating the correction graph. In the top row, from left to right, the phantoms are Pat63, Pat13, Pat47, Pat14 and Pat62. In the bottom row, from left to right, the phantoms are Pat61, Pat33, Pat21, Pat60 and Pat40

The patient´s CT data were acquired using a Siemens Symbia Intevo Bold System (Siemens Healthineers, Germany). The SPECT/CT acquisition was performed at about 24-h post injection as part of routine dosimetry workflow. The CT parameters were as follows: tube voltage of 110 kV; tube time–current 24 mAs reference; slice collimation 16 × 1.2 mm; and pitch 1.5. The CT images were reconstructed using the manufacturer`s implementation of the iterative reconstruction algorithm with an I31s kernel and a slice thickness of 5 mm. An overview of the lymph node phantoms is shown in Fig. 1.

### Segmentation and Design of the Lymph Node Phantoms

**Fig. 2:**
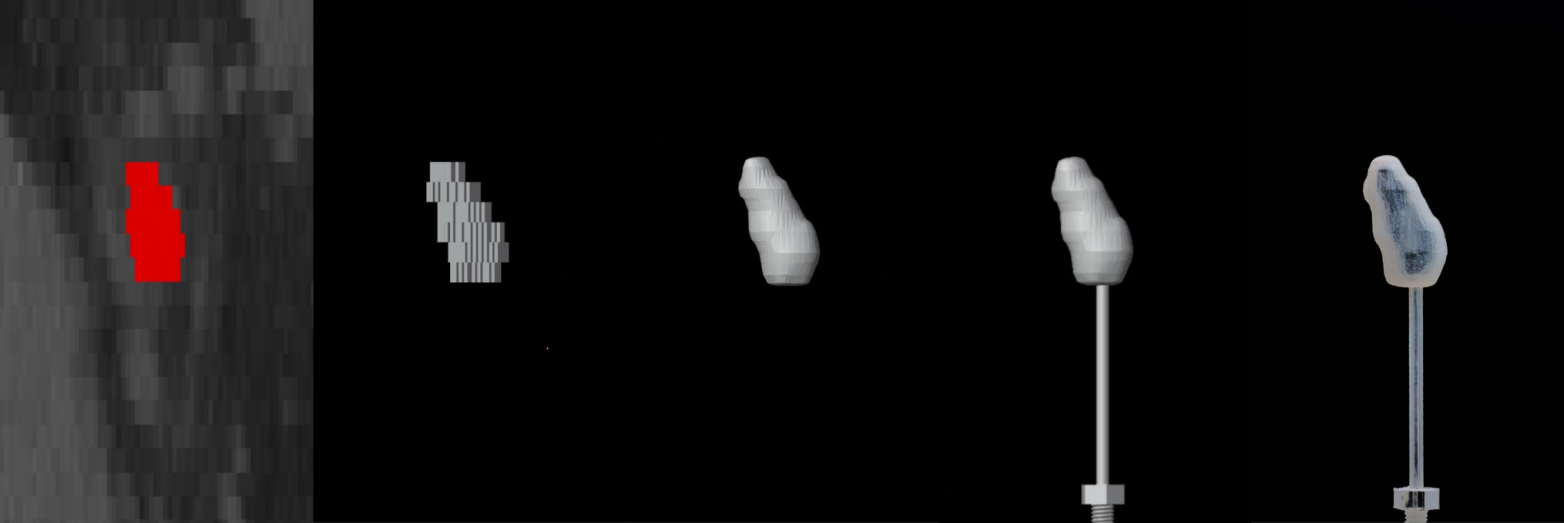
Manufacturing process of lymph node phantoms from segmentation on CT to the printed phantom, exemplified by Pat47

LNM were segmented from the patient`s CT data using ITK-Snap software (version 4.0.2). For this purpose, LNM were manually contoured voxel by voxel in the transverse slices of the CT images. The resulting segmentation was then exported as surface mesh in STL format and further processed in the CAD software Meshmixer (Version 3.5). In Meshmixer, the segmentation of LNM was smoothed using the “Smooth” function. This function was employed to generate a smoothed, anatomically realistic surface model from the voxelized segmentation. The smoothing mode “Shape Preservation” was applied in this process. The smoothing scale was set between 1 and 5, depending on the volume of LNM, with the smoothing factor set to 1 and “Constrain Rings” to 3. The smoothing scale parameter controls the intensity of the smoothing, where 0 represents no smoothing, and 100 represents the maximum possible smoothing. For very small LNM with low volumes, lower values were used to minimize the impact on model volume and to remain as close as possible to the original segmentation. Higher smoothing values were applied for models with larger volumes, as the effect on volume was less pronounced in these cases. To quantify whether CAD smoothing altered lesion volume, volumes of the original CT-based segmentations (ITK-Snap) were measured and compared with the volumes of the smoothed STL models (Meshmixer). A wall with a thickness of 1 mm was added around the segmentation to create a hollow structure. For fixation and filling of the models, a filling-connector was attached at the lower end. The connector has a total length of 50 mm and includes a threaded screw for attachment to the torso phantom. The connector contains a small hole through which the LNM-phantoms can be filled. The manufacturing process of the lymph node phantoms is shown in Fig. 2.

### Determination of the Surface-to-Volume Ratios (SA:V)

The lymph node phantoms' surface-to-volume ratio (SA:V) was determined after the previously described smoothing process in Meshmixer. For this purpose, the "Stability" module was used, which calculates both the surface area and the volume of the models.

### Printing of the Lymph Node Phantoms

All lymph node phantoms were produced using a Keyence Agilista-3200W 3D printer. The phantom walls were printed using the printer-specific Keyence material AR-M2, a rigid translucent polyacrylate resin, while the water-soluble Keyence material AR-S1 was used as support material during the printing process. After printing, the support material was thoroughly removed by repeatedly flushing the phantoms with water through the filling connector.

### SPECT/CT Measurement and Evaluation

For the phantom scans, five phantoms were placed in a torso phantom (similar to the body phantom NEMA-NU 2007, PTW-Freiburg, Freiburg, Germany) using a base plate. The placement was arranged so that each lymph node phantom maintained an equal distance from the wall to the center of the torso phantom. The ten printed lymph node phantoms were divided into two measurement series, each conducted with four different target-to-background ratios (TBR). Measurement series 1 included the phantoms P13, P14, P21, P33 and P47, while measurement series 2 included the phantoms P40, P60, P61, P62 and P63. The phantoms were all filled with the lutetium compound [^177^Lu]Lu-DOTATOC to prevent adhesion to the model walls, which occurs more frequently when using pure lutetium.

### Measurement Series 1

The lymph node phantoms were filled with a 216.8 kBq/ml concentration of [^177^Lu]Lu-DOTATOC. The background in the first scan remained cold, i.e., without activity. For the subsequent scans, the background was filled with 10.7, 21.6, and 42.5 kBq/ml, respectively. As a result, the actual target-to-background ratios for the planned 20:1, 10:1, and 5:1 were 20.7:1, 10.0:1, and 5.1:1, respectively.

### Measurement Series 2

The lymph node phantoms were filled with a 197.2 kBq/ml concentration of [^177^Lu]Lu-DOTATOC. The background in the first scan remained cold, i.e., without activity. For the subsequent scans, the background was filled with 10.6, 20.9, and 42.1 kBq/ml, respectively. As a result, the actual target-to-background ratios for the planned 20:1, 10:1, and 5:1 were 18.6:1, 9.4:1, and 4.6:1, respectively.

To facilitate the later segmentation of the reconstructed SPECT/CT images, the lymph node phantoms were additionally filled with CT contrast agent (Imeron 350, Bracco Imaging, Konstanz, Germany) in a 1:20 ratio. This slightly increased this compartment's Hounsfield units (HU) to approximately 362.25 HU.

### SPECT/CT Scanner

All phantom scans were performed using a clinical SPECT/CT system (Symbia Intevo Bold, Siemens Healthineers, Germany) with a medium-energy low-penetration (MELP) collimator. The scans were carried out with the following configurations: 180° detector configuration, 120 projections over 360° (3° sampling) in 60 views at 30 s per view, a projection matrix of 256 × 256 and a pixel spacing of 1.95 × 1.95 mm. The energy window was set between 187.56 to 229.24 keV, with a lower scatter window from 166.72 keV to 187.56 keV and an upper scatter window from 229.24 keV to 250.08 keV. To obtain quantitative values, the SPECT system was calibrated using Se-75 precision reference sources as part of the Siemens xSPECT Quant technology (Siemens). The CT parameters of the hybrid SPECT/CT acquisition were set as follows: tube voltage 130 kV; tube time–current 78 mAs reference; Siemens Care Dose 4D tube current modulations; slice collimation 0.6 × 9.6 mm and pitch 1.1. The CT images were reconstructed using the manufacturer’s recommended iterative reconstruction algorithm with the I31s kernel and a slice thickness of 1 mm. The SPECT projection data was reconstructed using the Siemens xSPECT Quant technology, based on an ordered-subset conjugate-gradient (OSCG) algorithm. Corrections were performed according to the manufacturer`s implementations. In brief, scatter radiation was corrected using a dual-energy window method and attenuation correction was performed based on CT. The reconstructed SPECT images had a matrix size of 256 × 256 with a voxel size of 1.95 × 1.95 × 1.95 mm. Four reconstructions were performed with 12, 24, 48, 72 iterations, all using one subset, resulting in fully quantitative SPECT data. The recovery coefficient (RC) for each lymph node phantom was determined from these reconstructions. The ratio of the concentrations obtained with SPECT to the actual concentrations (known from the phantom filling) was calculated. Finally, the activity was extracted using a Python script after manually segmenting the respective volumes in ITK-Snap.

### Single Volume-of-Interest (VOI) Method

The single VOI method calculates the activity of a structure based on a larger volume of interest surrounding the actual structure. This approach includes the activity within the structure itself and that which has been washed out from these structures due to the partial volume effect. Additionally, this method can include activity from the background within the VOI. For this, a VOI was placed around the visually observed uptake in the SPECT image using the MM Oncology workflow in the software Syngo.via (Version: VB60A, Siemens Healthineers, Germany).

### Multi VOI Method

To use the oversized VOI method, an enlarged VOI is first placed around the target structure to contain the entire activity of the source. Additionally, two concentric VOIs with increasing volume are placed around the initial VOI to include surrounding radioactivity. Assuming that the contribution of activity from the surrounding tissue increases linearly with the VOI size, the actual activity in the target region can be determined by extrapolating the activity against the VOI volume [27]. To apply this correction method, the images of the phantom scans were loaded with the MM Oncology workflow in Syngo.via (Version: VB60A, Siemens Healthineers, Germany), and three VOIs were manually placed around each lymph node phantom. The second and third VOIs correspond to twice and three times the volume of the first VOI, respectively. Using the VOI parameters in Syngo.via, the corrected activity of the structure can be calculated using formula (1) from the Volume (X) and activity within the VOIs (Y). Here, Y_X0_ denotes the y-intercept at X = 0 and represents the estimated true lesion activity at zero added background.

1 $${Activity (Y}_{X0})= \frac{\sum Y \times \sum {X}^{2}- \sum \left(X \times Y\right) \times \sum X}{3\sum {X}^{2}-{(\sum X)}^{2}}$$

### Single Target Correction (STC)

The single target correction (STC) method was developed for cases where only a single image region requires correction. STC operates on a voxel-based level and does not require the definition of background regions. The STC algorithm involves several steps, during which the target volume and the background are corrected voxel by voxel. Using STC requires precise segmentation of the target volume as well as knowledge of the point-spread function (PSF), which describes the extent of image blurring. After each correction step, a new image estimate is generated, serving as the basis for further iterations, thereby increasing correction accuracy. Compared to some other PVC approaches, an advantage of STC is that only a single region needs to be segmented, whereas other methods often require segmentation of the entire image. Furthermore, with accurately performed segmentation, STC provides voxel-level corrected images with improved edge sharpness, potentially enhancing the detection of tumor lesions [28]. The single target correction was applied to individual lymph node phantoms using the PETPVC toolbox developed by Thomas et al. [29]. For this purpose, the phantoms were manually segmented again on the CT image using ITK-snap. To approximate the point-spread function, three rod sources were placed in the same arrangement as the lymph node phantoms within the torso phantom, with one source each positioned along the X, Y, and Z axes. This setup allowed the approximate determination of the PSF in the X, Y, and Z directions at the position of the lymph node phantoms. The PSF values used were 17.30 mm, 17.77 mm, and 16.86 mm for the X, Y, and Z axes. The number of iterations was set to 10, which has been identified as optimal in other studies [30]. After STC correction, the recovery coefficient (RC) for each phantom was determined from the STC-corrected images using a Python script.

### Recovery Coefficient-Based Method

This method represents the first and simplest algorithm for correcting measurements by applying a correction factor known as the recovery coefficient (RC). It was initially introduced by Hofman et al. for a single region [21]. The RC is defined as the ratio of the measured activity concentration of an object to its actual concentration [26]. To use this method, phantom measurements are first conducted, examining known volumes of different sizes, shapes, or surface-area-to-volume ratios (SA:V). These phantoms can represent organs, tumors, or geometric shapes. The resulting phantom data are analyzed to calculate an RC for each volume/SA:V. The RC indicates how much activity is lost due to the object`s size. In practice, the RC determined for a specific object size can be used to correct measurements of similar objects, thereby improving the accuracy of the determined activity. We selected the NEMA IEC body phantom set to apply an RC-based method to our lymph node phantoms because the spheres in this phantom were standardized in shape and size, making this phantom widely used. The phantom is acrylic (PMMA) and contains six fillable spheres with inner diameters of 10 mm, 13 mm, 17 mm, 22 mm, 28 mm, and 37 mm. All scans were conducted using the same camera as the lymph node phantoms, with identical technical parameters and the same setup. Similar to the lymph nodes, scans were performed with four different target-to-background ratios. The spheres of the phantom were filled with a concentration of 202.5 kBq/ml of [^177^Lu]Lu-DOTATOC. The background during the first scan remained cold, without activity. For subsequent scans, the background was filled with concentrations of 10.1, 20.2, and 40.7 kBq/ml, resulting in actual ratios of 20.0:1, 9.9:1, and 4.9:1 for the initially planned 20:1, 10:1, and 5:1 acquisitions, respectively. Finally, the spheres were manually segmented on the CT using ITK-snap. The RC for each sphere was extracted using a Python script. From the RCs and the background ratio, a non-linear regression was used, the function used an exponential decay with plateau using a least squares optimization with no weighting, it is shown in formula (2). The parameter Y_m_ represents the lower asymptote (or plateau) of the function and was fixed for each fit according to the respective target-to-background ratio (TBR) of the measurement. In contrast, Y_0_​ denotes the intersection with the y-axis, and was derived using GraphPad Prism via non-linear regression. The rate constant $k$ determines the steepness of the curve (how rapidly the RC decreases from Y_0_​ to Y_M​_ as the SA:V increases).

2 $$Y={Y}_{M}-\left({Y}_{M}-{Y}_{0}\right)\times {e}^{-k\times x}$$

## Results

The results of the determination of the surface area and volume in Meshmixer are shown in Table 1. The calculated SA:V for the NEMA spheres were 1.62 to 6.00 cm^−1^ and for the 3D-printed lymph node phantoms 1.86 to 11.27 cm^−1^, as indicated in Table 1. With this range of SA:V, these phantoms represent different organs and smaller lesions, like kidneys [31] and up to small lymph node metastasis. The median in relative volume change from the lymph node phantoms versus the segmentation of the LNM was −10.5% (range −27.2% to −0.3%); lesion-wise values are provided in Supplementary Table 4.

**Table 1:** Volumes, surface areas, and surface-area-to-volume ratios (SA:V) of the lymph node and NEMA sphere phantoms used in this study

Phantom	Volume [cm^3^]	Surface area [cm^2^]	SA:V [cm^−1^]
Lymph node phantoms Measurement Series 1			
Pat 13	4.42	14.60	3.30
Pat 47	2.26	10.40	4.60
Pat 14	0.63	4.07	6.41
Pat 21	0.19	1.97	10.36
Pat 33	0.17	1.97	11.27
Lymph node phantoms Measurement Series 2			
Pat 61	23.71	44.18	1.86
Pat 63	8.80	24.12	2.73
Pat 62	1.73	9.58	5.53
Pat 60	0.20	2.03	10.07
Pat 40	0.19	1.92	9.79
NEMA sphere phantoms			
Sphere 1	26.52	43.00	1.62
Sphere 2	11.49	24.63	2.14
Sphere 3	5.57	15.20	2.73
Sphere 4	2.57	9.07	3.53
Sphere 5	1.15	5.30	4.62
Sphere 6	0.52	3.14	6.00

**Fig. 3:**
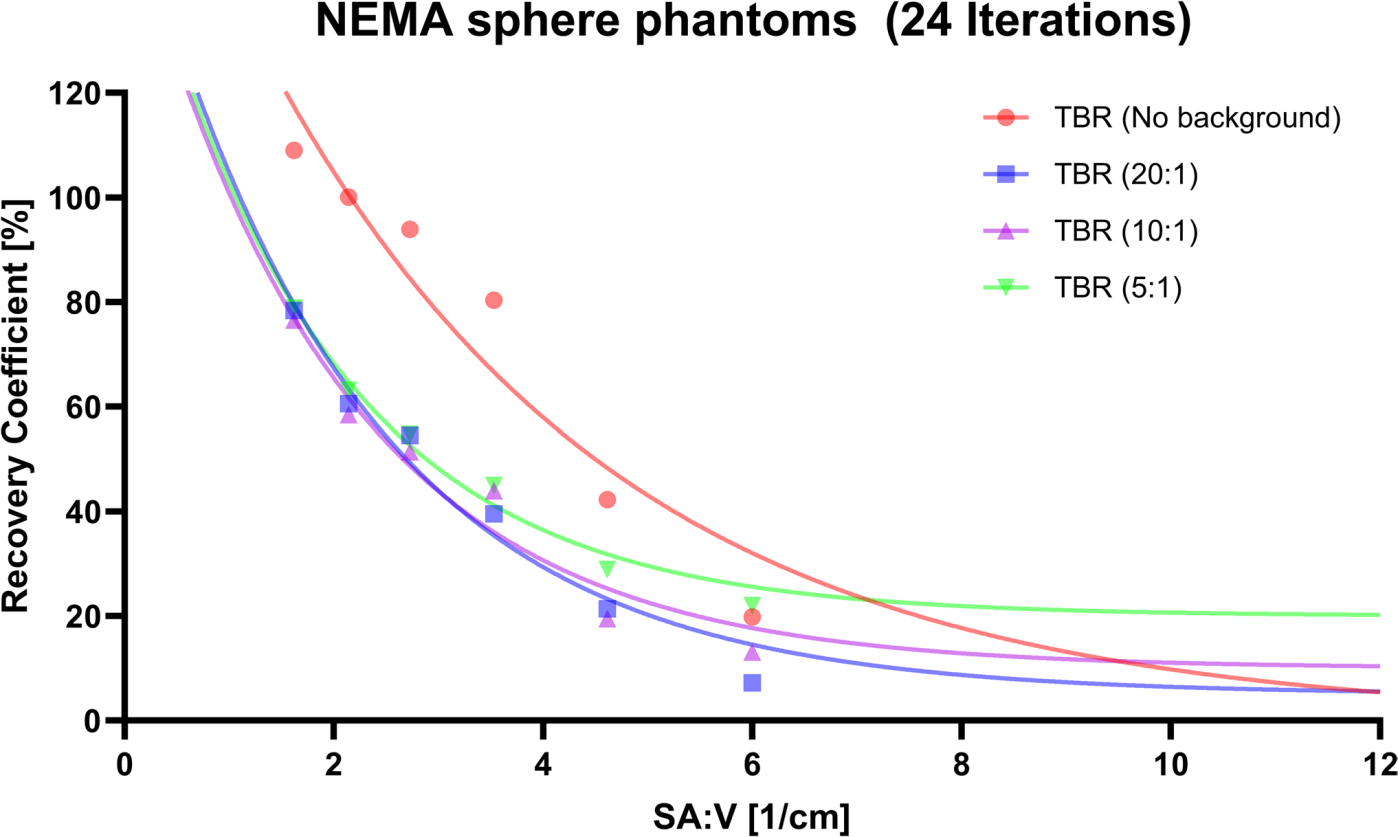
Exponential function fitted to the RC values of the NEMA sphere phantoms, converging to a plateau corresponding to the target-to-background ratio (TBR)

From the quantitative SPECT data, activity concentrations within the NEMA spheres and lymph node phantoms were determined using manual segmentations in ITK-SNAP and a custom Python script for activity quantification. Reconstructions were initially evaluated using different iteration numbers (12, 24, 48, and 72), and the resulting recovery coefficients (RCs) were calculated for three phantom setups with four different target-to-background ratios (TBR), as summarized in Supplementary Table 1 and 2. RC values ranged from 3.43% for the lymph node phantom with the highest surface-area-to-volume ratio in the no-background setting, up to 116.64% for the largest NEMA sphere. For the dosimetry dataset, reconstructions using 24 iterations were used consistently, as this setting reflects the established clinical standard in our department. Based on this standard configuration, partial volume corrections were derived and applied. The focus on this single reconstruction protocol was chosen to ensure consistency with clinical practice and to limit data complexity within the scope of the study. Figure 3 shows the extent of the partial volume effect in a graph with an exponential function fitted through the RC versus SA:V of the NEMA spheres individually for the different TBRs. The NEMA spheres can only show the range up to a SA:V of 6 cm^−1^, so a plateau was set to calculate higher SA:V structures. The plateau was set to the TBR of the individual measurement. They were set to 0%, 5%, 10%, and 20%, respectively for the measurements with no background, 20:1, 10:1, and 5:1.

In formula (2) the base formula for the exponential fit with a dedicated plateau is shown. Supplementary Table 3 shows the parameter for the different TBR. Y_M_ stands for the plateau which was set to a dedicated percentage and Y_0_ and k were calculated using the GraphPad Prism. The R^2^ for the graphs ranged from 0.91 to 0.98.

**Fig. 4:**
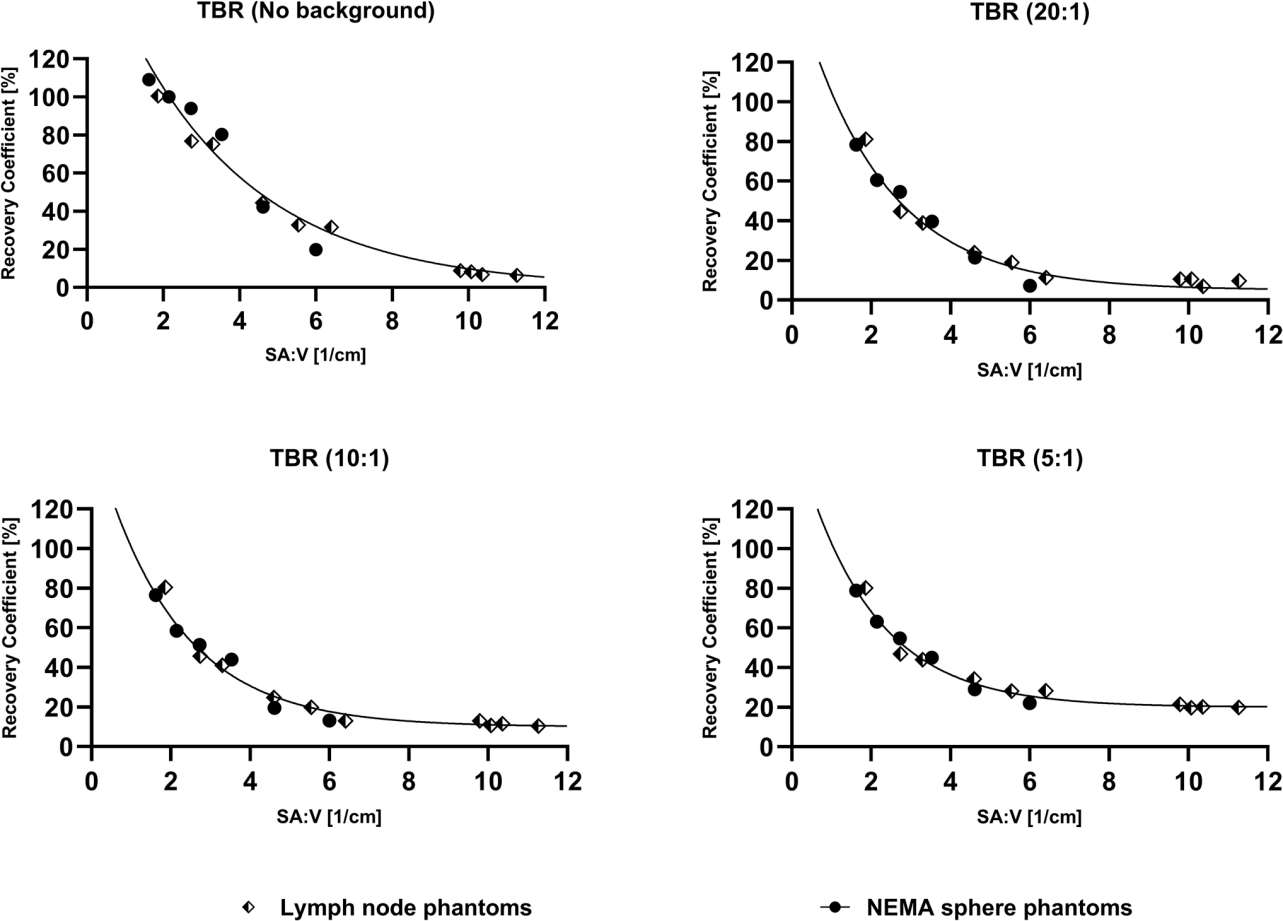
Fitted exponential function for the NEMA sphere phantoms (filled circles), combined with the lymph node phantoms (open diamonds) for validation of the graph, shown for all four TBR’s with 24 iterations

To verify the 4 different graphs the images of the lymph node phantoms were evaluated and the deviation to these graphs were calculated. Figure 4 shows the graphs together with the lymph node phantoms. The deviation from the RC’s of the lymph node phantoms to the exponential graph for the different TBR were 92.1 ± 10.2%, 120.8 ± 30.9%, 101.1 ± 10.7% and 102.1 ± 7.8% for the infinity, 20:1, 10:1 and 5:1 TBR’s respectively.

For the verification and comparison of the exponential fit, the 10:1 TBR function was used and compared with other post-reconstruction partial volume correction methods. The results for this are shown in Table 2. The RC´s without correction for the partial volume effect ranged from 10.37% to 80.42%. After applying the exponential fit (RC-based correction), the RCs improved to a range of 79.88% to 116.70%, with a mean of 101.15 ± 10.15% SD. The other correction methods resulted in mean RCs of 256.09 ± 111.32% SD (range 92.01% to 495.00%) for RC (single VOI), 57.86 ± 42.09% SD (range 12.35% to 150.20%) for RC (multi VOI), and 80.03 ± 36.80% SD (range 29.67% to 152.85%) for RC (STC).

**Table 2:** The uncorrected RC and the RC calculated using different correction methods for the lymph node phantoms with measurements at a TBR of 10:1

Phantom	RC (orig.) [%]	RC (RC-based) [%]	RC (Single VOI) [%]	RC (Multi VOI) [%]	RC (STC) [%]
Pat 13	41.03	104.64	141.40	88.94	129.56
Pat 47	24.78	97.64	221.02	101.21	95.52
Pat 14	13.04	79.98	130.93	20.13	58.82
Pat 21	11.57	106.22	289.67	24.67	86.82
Pat 33	10.37	98.11	358.56	6.84	30.50
Pat 61	80.42	115.96	145.75	117.62	145.25
Pat 63	45.75	94.31	190.91	116.16	100.70
Pat 62	19.81	100.62	239.25	61.44	58.38
Pat 60	10.74	97.32	348.66	18.89	64.95
Pat 40	13.05	116.70	494.77	22.66	29.81
Average	27.06	101.15	256.09	57.86	80.03
Min	10.37	79.88	92.01	12.35	29.67
Max	80.42	116.70	495.00	150.20	152.85
Standarddeviation	22.67	10.15	111.32	42.09	36.80

## Discussion

Investigating the partial volume effect typically requires phantom imaging, as phantoms provide a gold standard against which PVE correction methods can be validated [27, 31, 32]. The SPECT literature widely employs stylized phantoms, particularly spheres, as reference objects for this purpose, since phantom imaging and tomographic reconstruction are often carried out analogously to patient imaging [33, 34].

However, not only the size but also the shape of an object significantly affects the PVE [22]. In more complex or application-specific scenarios, a reasonable alternative is the development of application-specific phantoms. Therefore, 3D-printed phantoms like those used in this study may offer a more accurate basis for evaluating and correcting PVE-related errors [33, 35].

The surface-area-to-volume ratio, central to our RC-correction method, reflects structural compactness more accurately than volume alone. Since PVE predominantly affects boundary regions, SA:V is a critical parameter in determining the magnitude of the effect. Previous studies have shown that structures with a high SA:V ratio are more susceptible to spill-over effects due to the larger proportion of voxels near their surface [22]. This was also demonstrated in kidney cortex phantoms, where elevated SA:V values corresponded to pronounced partial volume effects [31].

In our study, lymph node phantoms exhibited SA:V ratios that were on average 20.5% higher than those of spheres with equivalent volume, with differences ranging from 10.5% to 37.3%. This underscores the importance of considering shape-specific factors when applying RC-based corrections. Applying standard RC graphs to non-spherical structures may lead to systematic overestimation, highlighting the need for geometry-aware correction strategies. Future studies should further investigate shape-dependent correction models to improve quantitative accuracy in dosimetry.

The importance of partial volume correction for small structures is evident in our results. Recovery coefficients for the lymph node phantoms varied widely, ranging from 10.4% in the smallest to 82% in the largest structure at a target-to-background ratio of 10:1. These deviations are substantially higher than those reported for larger organs such as the kidneys [31]. In dosimetry, such errors would translate directly into proportional inaccuracies in absorbed dose estimations, demonstrating that omission of PVC for lymph node metastases could lead to clinically relevant miscalculations.

Using the NEMA IEC body phantom, we established an exponential relationship between SA:V and RC, with R^2^ values ranging from 0.91 to 0.98 across measurements at four different TBRs. To account for small-volume behavior, we incorporated TBR as a defined plateau within the correction graphs. This allowed correction for high-SA:V structures not adequately represented by the NEMA spherical phantoms. As a result, instead of a linear trend as observed in the NEMA spheres, an exponential correlation emerged.

The linear behavior of large volumes in relation to FWHM has been extensively discussed in previous studies [31, 36, 37]. Our findings are consistent with this, but also extend the concept to smaller and more complex geometries. This aligns with observations by Marquis et al. [38], who demonstrated that RC curves for differently shaped volumes (spheres and ellipsoids) can be unified by expressing them as a function of (V/SA)/FWHM. For larger structures (RC > 0.7), a relatively uniform trend persists, while smaller and less compact structures show increased nonlinearity, a pattern consistent with our data (Fig. 3).

Using our SA:V-based correction curves, RCs of lymph node phantoms were on average 101.15 ± 10.15% (mean ± SD) of the ground truth. Compared to the uncorrected activity estimates and other correction methods, this approach yielded results significantly closer to the true activity values. Statistical significance was assessed using a paired t-test and Wilcoxon signed-rank test, with p-values < 0.05 considered significant.

The RC-based method showed statistically significant improvements over uncorrected RCs (*p* = < 0,001), single-VOI (*p*= 0.002), and multi-VOI methods (*p*= 0.012). These findings were corroborated by Wilcoxon signed-rank tests (p = 0.002, p = 0.002, and p = 0.037, respectively). No significant difference was found compared to the STC method (*p* = 0.111, t-test; *p*= 0.160, Wilcoxon), likely due to the high variability in STC results. While STC achieved comparable average accuracy in some cases, the RC-based correction consistently demonstrated lower variance and more stable performance, underscoring its potential for robust partial volume correction in clinical workflows. The alternative correction methods produced average RCs of 256.09 ± 111.32%, 57.86 ± 42.09%, and 80.03 ± 36.80% for the single-VOI, multi-VOI, and STC methods, respectively.

Several limitations of this study should be acknowledged. First, the number of lymph node phantoms was relatively small. However, given the high statistical significance of our results, we do not expect the observed trends to change substantially with larger samples. Moreover, our data suggest that the relationship between RC and SA:V depends on TBR. In clinical imaging, TBR is often unknown, which may introduce uncertainty when applying SA:V-based correction.

Determining TBR in clinical practice is challenging. Based on prior work in PET and SPECT imaging [39, 40], a pragmatic approach could involve placing a target ROI on the lesion and a background ROI in a homogeneous reference region. Depending on the background region selected (e.g., vascular regions or healthy soft tissue), TBR values may vary significantly and influence quantification [28]. Another factor affecting TBR is the timing of image acquisition. Early post-injection scans may result in artificially high TBRs due to residual circulating activity, while delayed imaging tends to stabilize this ratio.

In Lu-177 dosimetry, where scans are typically performed beyond 24 h, TBR values may naturally increase, reducing the impact of background activity. Nonetheless, standardizing the imaging time could further improve consistency, as previously suggested for FDG-PET imaging [41].

Validation of the RC-correction method was performed using 3D-printed lymph node phantoms generated from segmented CT data and fabricated via inkjet printing. While CT resolution and segmentation voxel size impose some limitations on shape fidelity, this did not influence our results, as the printed geometries themselves served as ground truth. CAD model smoothing can affect SA and volume estimates but also leads to more realistic shapes compared to the voxel-wise representation of the previous segmentations; however, this effect is minimized by high-resolution printing and consistent processing.

In clinical settings, precise SA:V estimation is currently not feasible with most SPECT/CT software, as these systems typically lack tools for surface area measurement. Therefore, routine clinical application of SA:V-based correction would require integration of advanced segmentation and analysis tools. Importantly, such limitations are less critical in larger structures, where PVE is less pronounced. Overall, our findings support the use of SA:V-based correction strategies in quantitative Lu-177 SPECT/CT imaging, particularly for small, irregularly shaped lesions such as lymph node metastases.

## Conclusions

The low recovery coefficients obtained for lymph node metastases suggest that Lu-177-dosimetry is in these structures grossly inaccurate without partial volume effect correction. For this purpose, we established a surface-area-to-volume to RC curve-based partial volume effect correction using the NEMA IEC body phantom. This method led to a considerable reduction in PVE-related errors when applied to patient-specific lymph node phantoms. Compared to other post-reconstruction correction approaches, the SA:V/RC-based correction yielded more consistent and quantitatively accurate results.

## Supplementary Information

Below is the link to the electronic supplementary material.

ESM 1 (DOCX 2.83 MB)

## Data Availability

All research data and computer codes are available from the corresponding author upon request.
