# Supplementary material for: Estimation and Correction of the Partial Volume Effect Using Personalized Phantoms of Lymph Node Metastases in Lutetium 177 SPECT/CT Dosimetry
Source: Mol Imaging Biol. 2026 Apr 29;28(3):470–80. doi: 10.1007/s11307-026-02103-x (PMC13337594; doi:10.1007/s11307-026-02103-x)

**Electronic Supplementary Material**

**Estimation and correction of the partial volume effect using personalized phantoms of lymph node metastases in Lutetium 177 SPECT/CT dosimetry**

**Journal: Molecular Imaging and Biology**

J. R. Hinz^1 2^, T. Kuwert^1 2^, M. Beck^1 2^, P. Ritt ^2^, A. Grings^1 2^

1. Department of Nuclear Medicine, Uniklinikum Erlangen, Erlangen, Germany
2. Chair of Nuclear Medicine, Friedrich-Alexander-Universität Erlangen-Nürnberg (FAU)

**Corresponding author:**

Andreas Grings

Ulmenweg 18

91054 Erlangen, Germany

Tel: +49 9131 85 47064

Email: Andreas.Grings@uk-erlangen.de

Online Resource 1: Recovery coefficients (RCs) for the Phantoms in percent [%], four different target to background ratios and four different SPECT reconstruction iteration numbers (12 and 24).

| Phantom | 12 Iterations | | | | 24 Iterations | | | |
| --- | --- | --- | --- | --- | --- | --- | --- | --- |
|  | Infi | 20:1 | 10:1 | 5:1 | Infi | 20:1 | 10:1 | 5:1 |
| Lymph node phantoms Measurement Series 1 | | | | | | | | |
| Pat 13 | 59.92 | 32.69 | 34.06 | 38.60 | 75.16 | 38.87 | 41.03 | 43.98 |
| Pat 47 | 33.31 | 20.36 | 21.39 | 30.43 | 44.35 | 23.93 | 24.78 | 34.28 |
| Pat 14 | 22.23 | 10.27 | 12.26 | 25.98 | 31.64 | 11.25 | 13.04 | 28.28 |
| Pat 21 | 4.05 | 6.23 | 10.93 | 19.61 | 6.73 | 6.87 | 11.57 | 20.05 |
| Pat 33 | 3.43 | 8.15 | 10.14 | 19.69 | 6.39 | 9.64 | 10.37 | 19.81 |
| Lymph node phantoms Measurement Series 2 | | | | | | | | |
| Pat 61 | 95.00 | 74.26 | 73.53 | 73.17 | 100.49 | 81.16 | 80.42 | 80.05 |
| Pat 63 | 57.58 | 37.85 | 39.55 | 42.03 | 76.79 | 44.72 | 45.75 | 46.84 |
| Pat 62 | 21.17 | 15.76 | 18.42 | 27.07 | 32.70 | 18.99 | 19.81 | 28.18 |
| Pat 60 | 3.64 | 9.66 | 10.28 | 20.19 | 8.18 | 10.60 | 10.74 | 19.67 |
| Pat 40 | 3.69 | 9.37 | 13.23 | 21.75 | 8.73 | 10.58 | 13.05 | 21.48 |
| NEMA sphere phantoms | | | | | | | | |
| Sphere 1 | 94.44 | 70.00 | 67.92 | 70.13 | 109.02 | 78.34 | 76.55 | 78.83 |
| Sphere 2 | 75.74 | 50.89 | 49.36 | 53.33 | 100.02 | 60.56 | 58.45 | 63.15 |
| Sphere 3 | 67.02 | 45.90 | 43.69 | 47.32 | 93.88 | 54.50 | 51.42 | 54.70 |
| Sphere 4 | 53.22 | 33.43 | 37.33 | 39.63 | 80.31 | 39.52 | 43.91 | 44.98 |
| Sphere 5 | 24.94 | 18.12 | 17.28 | 25.80 | 42.22 | 21.40 | 19.48 | 28.92 |
| Sphere 6 | 9.60 | 6.68 | 11.91 | 20.52 | 19.84 | 7.23 | 13.11 | 21.97 |

Online Resource 2: Recovery coefficients (RCs) for the Phantoms in percent [%], four different target to background ratios and four different SPECT reconstruction iteration numbers (48 and 72).

| Phantom | 48 Iterations | | | | 72 Iterations | | | |
| --- | --- | --- | --- | --- | --- | --- | --- | --- |
|  | Infi | 20:1 | 10:1 | 5:1 | Infi | 20:1 | 10:1 | 5:1 |
| Lymph node phantoms Measurement Series 1 | | | | | | | | |
| Pat 13 | 92.68 | 46.51 | 49.19 | 49.95 | 96.18 | 50.87 | 54.09 | 53.48 |
| Pat 47 | 64.19 | 28.43 | 28.74 | 38.74 | 70.89 | 31.07 | 31.03 | 41.27 |
| Pat 14 | 49.68 | 12.44 | 14.20 | 30.73 | 55.66 | 13.02 | 14.98 | 31.92 |
| Pat 21 | 11.97 | 8.07 | 12.84 | 21.07 | 14.02 | 8.97 | 13.81 | 21.88 |
| Pat 33 | 11.02 | 11.98 | 10.91 | 20.29 | 12.88 | 13.43 | 11.36 | 20.57 |
| Lymph node phantoms Measurement Series 2 | | | | | | | | |
| Pat 61 | 104.43 | 86.71 | 86.66 | 85.96 | 106.49 | 89.28 | 89.48 | 88.61 |
| Pat 63 | 90.28 | 51.49 | 53.41 | 52.84 | 98.40 | 55.26 | 57.93 | 56.63 |
| Pat 62 | 45.20 | 22.69 | 21.48 | 29.48 | 58.57 | 24.91 | 22.28 | 30.10 |
| Pat 60 | 12.07 | 11.63 | 11.42 | 18.52 | 14.80 | 12.30 | 11.89 | 17.43 |
| Pat 40 | 13.19 | 11.99 | 12.17 | 20.35 | 16.72 | 13.01 | 11.27 | 19.10 |
| NEMA sphere phantoms | | | | | | | | |
| Sphere 1 | 113.14 | 85.82 | 84.68 | 87.05 | 116.64 | 89.48 | 88.65 | 91.21 |
| Sphere 2 | 108.35 | 70.39 | 68.09 | 73.85 | 111.58 | 75.79 | 73.35 | 79.90 |
| Sphere 3 | 105.89 | 63.77 | 59.82 | 62.37 | 113.15 | 69.18 | 64.46 | 66.62 |
| Sphere 4 | 96.23 | 46.54 | 51.37 | 50.88 | 112.93 | 50.89 | 55.85 | 54.29 |
| Sphere 5 | 56.22 | 25.33 | 22.17 | 32.80 | 84.50 | 27.93 | 24.07 | 35.25 |
| Sphere 6 | 28.00 | 7.55 | 14.52 | 23.87 | 45.89 | 7.55 | 15.21 | 24.94 |

Online Resource 3: Model parameters of the exponential SA:V/RC fit used for partial volume correction.

| TBR | Y_m_ (set by user) [%] | Y_0_ [%] | k | R^2^ |
| --- | --- | --- | --- | --- |
| **Infi** | 0 | 189.7 | 0.29 | 0.91 |
| **20:1** | 5 | 164.6 | 0.46 | 0.96 |
| **10:1** | 10 | 158.8 | 0.49 | 0.95 |
| **5:1** | 20 | 161.8 | 0.53 | 0.98 |

Online Resource 4: Pre- and post-smoothing lesion volumes and relative volume change (%) induced by CAD smoothing.

| Phantom | Pre-smoothed volume (cm³) | Post-smoothed volume (cm³) | Relative change (%) |
| --- | --- | --- | --- |
| Lymph node phantoms Measurement Series 1 | | | |
| Pat13 | 4.687 | 4.420 | -5.7 |
| Pat47 | 2.456 | 2.260 | -8.0 |
| Pat14 | 0.696 | 0.630 | -9.5 |
| Pat21 | 0.215 | 0.190 | -11.5 |
| Pat33 | 0.234 | 0.170 | -27.2 |
| Lymph node phantoms Measurement Series 2 | | | |
| Pat61 | 23.779 | 23.710 | -0.3 |
| Pat63 | 9.036 | 8.800 | -2.6 |
| Pat62 | 1.955 | 1.730 | -11.5 |
| Pat60 | 0.253 | 0.200 | -20.9 |
| Pat40 | 0.248 | 0.190 | -23.4 |

Online Resource 5: Representative CT (top) and reconstructed SPECT (bottom) images of the patient-specific lymph node phantoms for the four target-to-background conditions (No BG, 20:1, 10:1, 5:1). Left column shows Measurement series 1 and right column Measurement Series 2. A CT contrast agent was used to improve the visibility of the phantom structures in the CT images.


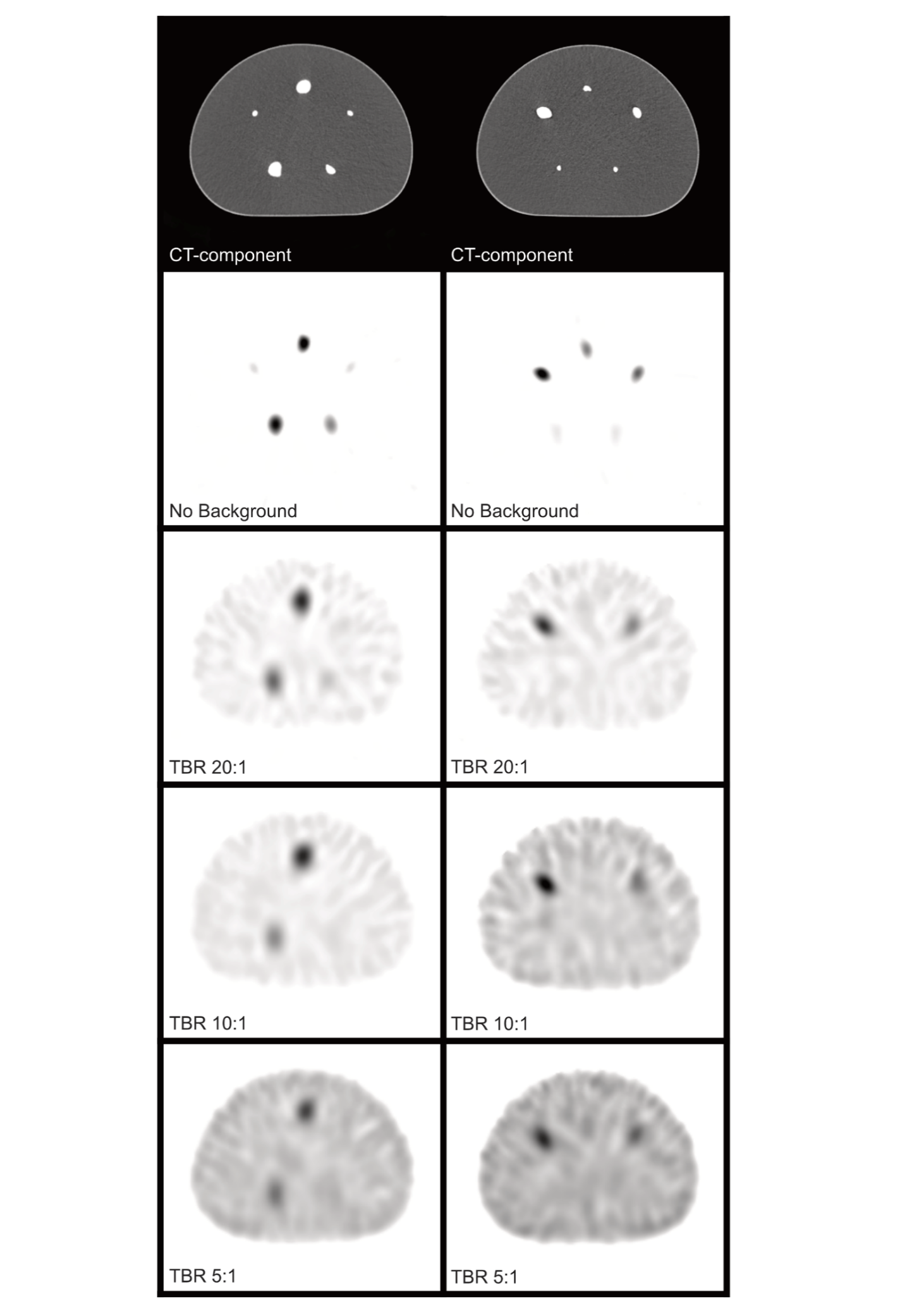


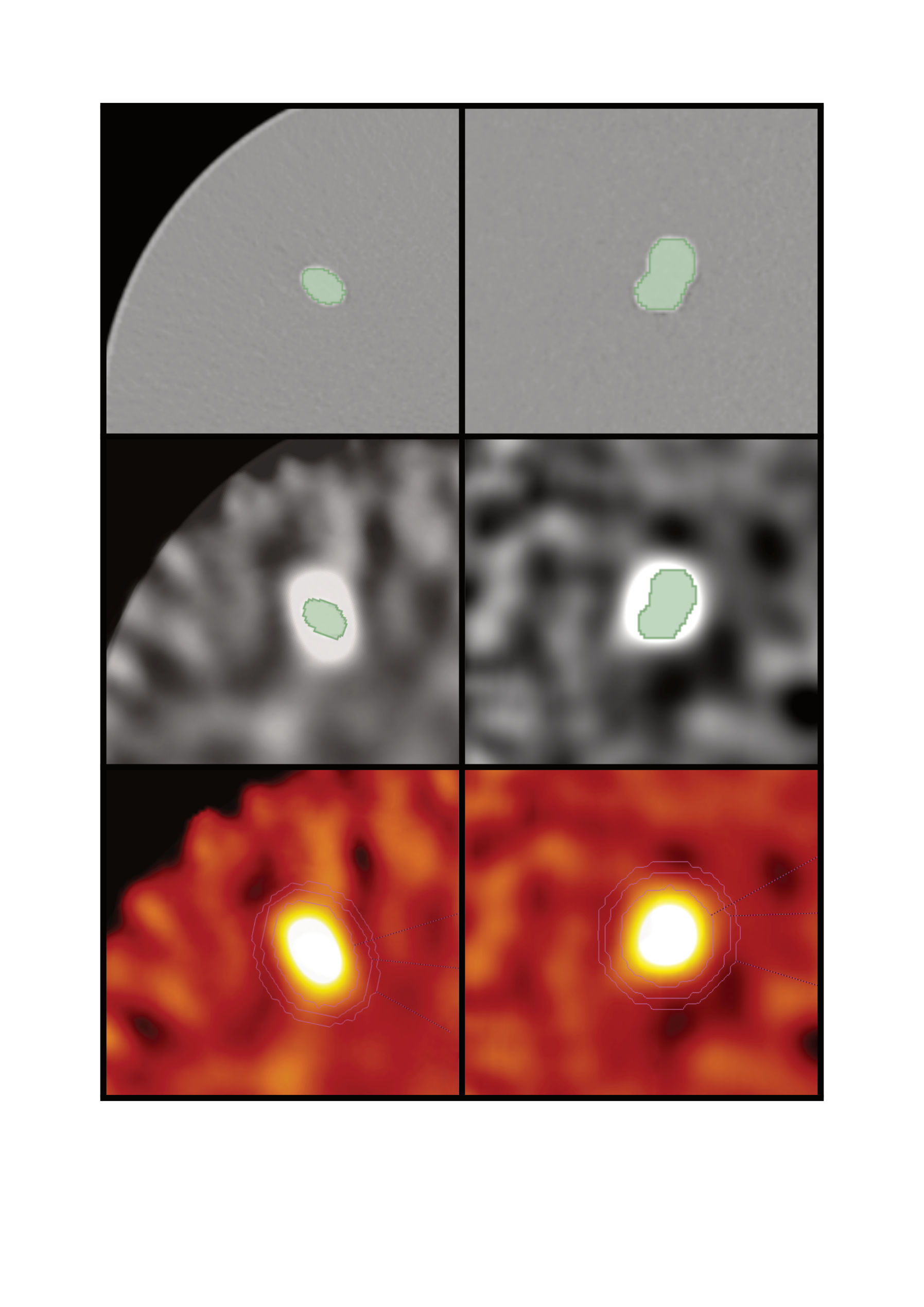


Online Resource 6: ROI/VOI definition for phantom P13 (TBR 10:1). CT-based target segmentation (green) shown on CT (top) and overlaid on reconstructed SPECT (middle); syngo.via screenshots illustrate the VOI-based approaches with concentric VOIs (bottom) in two views.

Online Resource 7: Representative cut-line profile for phantom P13 at a target-to-background ratio of 10:1. The profile was derived from the reconstructed image data to illustrate the edge behavior of the phantom. In an idealized activity distribution, the activity would briefly drop to zero while crossing the 1-mm cold phantom wall; however, this contribution is expected to be minor relative to the limited spatial resolution of ^177^Lu-SPECT.


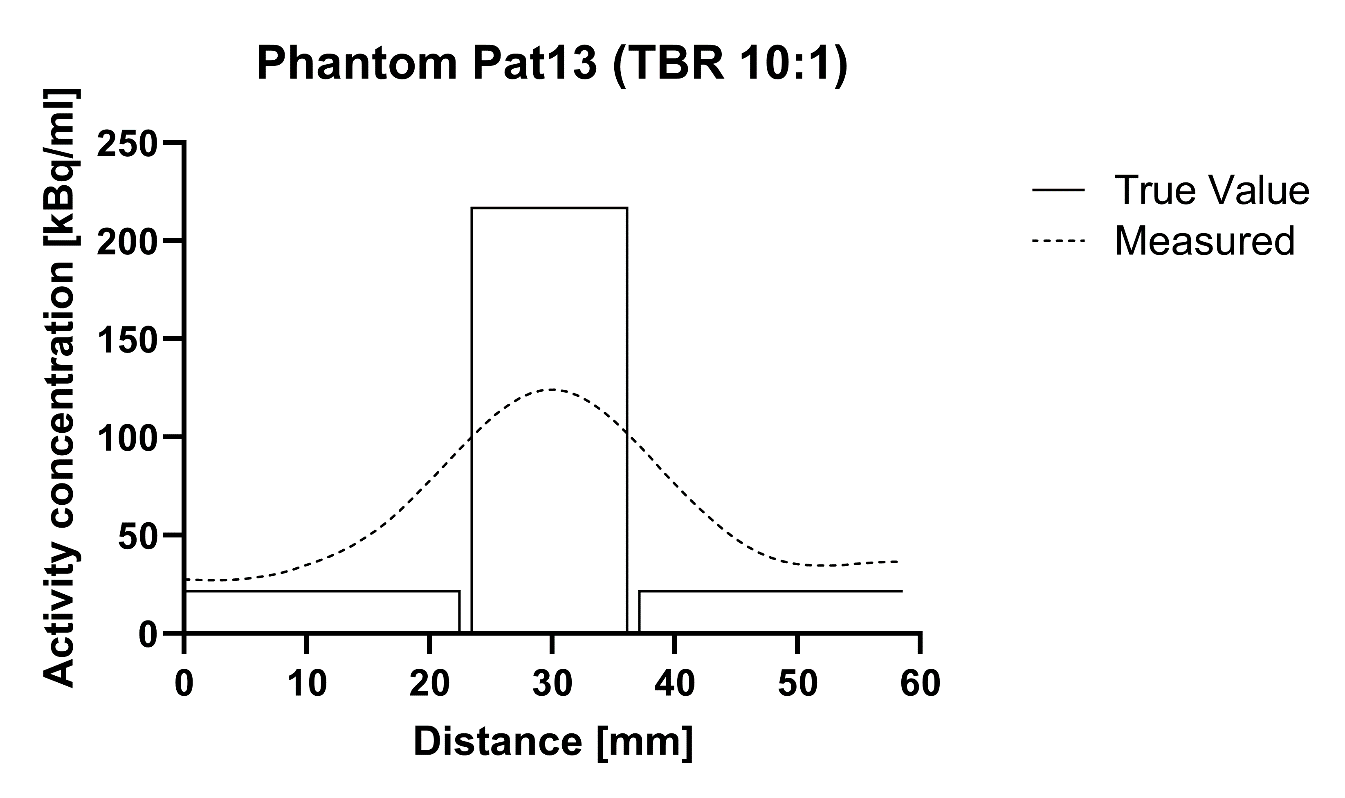

Supplement: Supplementary file 1 — (DOCX 2.83 MB) [file 11307_2026_2103_MOESM1_ESM.docx]
